# Linking health system inputs, processes and outputs to identify medical deserts in Serbia: Stefan Mandić-Rajčević

**DOI:** 10.1093/eurpub/ckac131.284

**Published:** 2022-10-25

**Authors:** S Mandić-Rajčević, M Šantrić-Milićević

**Affiliations:** 1 University of Belgrade, Faculty of Medicine, Institute of Social Medicine, Belgrade, Serbia; 2 University of Belgrade, Faculty of Medicine, School of Public Health, Belgrade, Serbia

## Abstract

**Background:**

Medical deserts (regions where the population does not have adequate access to health care) indicate the failure of the health system to achieve the goals of improving the health of the population. Identifying medical deserts (MD) is far from simple. The aim of this study is to describe the approach to identification of medical deserts in Serbia using indicators for the health system inputs, processes and outputs.

**Methods:**

We investigated the basic healthcare-related medical deserts using the indicators of primary health care centers’ inputs (accessibility: annual number of patients per physician), processes (performance: annual workload of patient visits per physician) and outputs (unmet needs: percentage of patients unable to access health services) in all 25 Serbian districts in 2020, using data of the Health Statistical Yearbook of the Republic of Serbia and the National Patient Satisfaction Survey of the Institute of Public Health. We developed a Multiple Criteria Scoring System (MCSS) incorporating the weighting and scoring of accessibility and performance for four types of physicians (general practitioners, pre-school pediatricians, youth pediatricians, and gynecologists) and five dimensions of unmet needs (financial reasons, waiting times, lack of personal time, long-distance and COVID-19). MCSS final scores 0 (none) - 100% (MDs on all indicators) are assessed using the regulatory norms.

**Results:**

MDs partially overlap by different criteria: accessibility, 4-10 districts; performance, three districts; unmet needs: 2-5 districts. Top five medical deserts identified according to the MCSS are Mačvanski, Šumadijski, Moravički, Srednjebanatski, and Podunavski district.

**Conclusions:**

Serbia has at least one MD per administrative region according to the objective normative indicators and patients’ subjective experiences. The study findings can be used to inform district stakeholders on how to use health workforce policy and planning to address medical deserts.

**Key messages:**

• MCSS indicates potential medical deserts in 20% of all districts in the Republic of Serbia.

• Evidence on poor health workforce accessibility and performance in light of the patient unmet healthcare needs could be used to inform stakeholders on medical deserts in the country.

